# Olaparib monotherapy or combination therapy in lung cancer: an updated systematic review and meta- analysis

**DOI:** 10.3389/fonc.2025.1505889

**Published:** 2025-03-24

**Authors:** Sajjad Hajihosseini, Ehsan Emami, Seyed Amirali Zakavi, Parnia Jochin, Mehregan Shahrokhi, Sahar Khoshravesh, Mitra Goli, Mohaddeseh Belbasi, Gisou Erabi, Niloofar Deravi

**Affiliations:** ^1^ Student Research Committee, Tehran University of Medical Sciences, Tehran, Iran; ^2^ Students Research Committee, School Of Medicine, Ardabil University of Medical Sciences, Ardabil, Iran; ^3^ School of Medicine, Shiraz University of Medical Sciences, Shiraz, Iran; ^4^ School of Medicine, Shahid Beheshti University of Medical Sciences, Tehran, Iran; ^5^ Department of Medical-Surgical Nursing, School of Nursing and Midwifery, Shahid Beheshti University of Medical Sciences, Tehran, Iran; ^6^ Students Research Committee, School of Pharmacy, Zanjan University of Medical Sciences, Zanjan, Iran; ^7^ Student Research Committee, Urmia University of Medical Sciences, Urmia, Iran; ^8^ Student Research Committee, Shahid Beheshti University of Medical Science, Tehran, Iran

**Keywords:** olaparib, lung cancer, NSCLCs, gefitinib, durvalumab

## Abstract

**Background and aims:**

Impaired double strand DNA repair by homologous repair deficiency (HRD) leads to sensitivity to poly ADP ribose polymerase (PARP) inhibition. A subset of non-small cell lung cancers (NSCLCs) harbour impaired DNA double strand break repair. This study aims to investigate meta-analysis on the olaparib monotherapy or combination therapy in lung cancer.

**Methods:**

A comprehensive search was conducted in Pubmed, Scopus and Google Scholar data bases up to August 13, 2023 related articles were extracted title, abstract and full text of articles were screened. The quality included articles were assessing the data was extracted and hence analysis.

**Results:**

After screening 5208 articles, 9 were selected for final review based on relevance to the topic. Olaparib monotherapy increased progression free survival (PFS) level [ES= 7.76; 95% CI= 0.16 to 1.36; P=0.208]. Olaparib maintenance therapy increased PFS compared to placebo in platinum-sensitive NSCLC patients [ES= 0.9; 95% CI= 0.9 to 0.9]. Combination therapy with durvalumab and olaparib decreased PFS level compared to the olaparib group [ES=6.07; 95% confidence interval (95% CI) = 0.67 to 11.46; P=0.000]. Adding gefitinib to olaparib decreased PFS compared to olaparib only group, significantly (ES=3.39; 95% CI=-0.78 to 7.56; P=0.609).

**Conclusions:**

Our study demonstrated olaparib as monotherapy can increase the PFS of patients with lung cancer, but the combination of olaparib and gefitinib or the combination of olaparib plus durvalumab couldn’t have a significant effect. According to the high heterogeneous rate of studies further large-scale randomized control trials are still required to progress association.

**Systematic Review Registration:**

Open Science Framework (OSF).

## 1 Introduction

Lung cancer remains the most common cancer site in men, accounting for 17% of all new cancer diagnoses and a staggering 23% of cancer-related deaths (1). The vast majority of lung cancers, approximately 80-85%, are classified as non-small cell lung cancer (NSCLC), which encompasses several histological subtypes including squamous cell carcinoma, adenocarcinoma, and large-cell carcinoma (2). Also, small cell lung cancer (SCLC) ranks as the sixth most common cause of cancer-related deaths, contributing to approximately 13–15% of all lung cancer cases (3, 4).

Poly (ADP-ribose) polymerase (PARP) enzymes constitute a family of nuclear enzymes that are responsible for identifying and repairing single-strand breaks in DNA (5–7). The primary function of PARP involves the poly-ADP ribosylation of essential chromatin components and various proteins that are integral to the DNA repair process (8). PARP1, in particular, has the ability to relax chromatin structure, thereby allowing DNA repair factors to access the damaged sites more effectively (9). Given the crucial role of PARP enzymes in DNA repair, PARP inhibitors have emerged as a promising avenue of research for the treatment of lung cancer, particularly NSCLC (10). In a study conducted by Byers et al. in 2012, it was found that SCLC cell lines exhibited significantly higher levels of PARP1 protein expression compared to NSCLC lines (11).

Numerous preclinical investigations have suggested that PARP inhibitors possess the ability to heighten the sensitivity of SCLC cells to a range of chemotherapeutic agents (12). Byers et al. were the first to report that incorporating olaparib into the standard chemotherapy regimen of cisplatin and etoposide enhanced the anti-tumor effects in SCLC (11). As another illustrative example, research conducted by Murai et al. revealed that the PARP inhibitor talazoparib enhances the cytotoxic effects of the DNA-alkylating agent temozolomide in cancer cells (12). Lallo et al. demonstrated that combining the PARP inhibitor (Olaparib) with the Wee1 kinase inhibitor (adavosertib) significantly enhances the effectiveness of olaparib as a single agent in patient-derived xenografts of SCLC (13). Additionally, an abstract study by Gay et al. highlighted a synergistic effect between an ataxia telangiectasia and Rad3 related (ATR) kinase inhibitor and olaparib, which resulted in increased cytotoxicity in SCLC cell lines. Collectively, these findings underscore the potential of a combinatorial approach as a promising therapeutic strategy for integrating PARP inhibitors into the treatment of SCLC (14).

But in clinical studies, a case report by Lin in 2024 presents a novel treatment approach involving the combination of osimertinib and olaparib for the management of concurrent lung and ovarian cancers. The authors describe two potential treatment approaches with this combination: an alternating schedule or a short-term concurrent administration (15). Maintenance therapy with the combination of durvalumab and olaparib in patients with metastatic NSCLC couldn’t demonstrate a statistically significant improvement in progression free survival (PFS) compared to durvalumab monotherapy; The length of time during and after the treatment of a disease, such as cancer, that a patient lives with the disease but it doesn’t get worse. However, a numerical improvement in PFS was observed with the combination regimen (16, 17). The results from a study by Fennell in 2022 indicate that while the primary endpoint of PFS was numerically longer in the olaparib treatment arm compared to the control group, this difference couldn’t meet the threshold for statistical significance. This suggests that PARP inhibitor monotherapy olaparib, may have the potential to achieve meaningful tumor control in chemosensitive NSCLC patients (18). Reduced levels of BRCA1 mRNA have been associated with longer PFS in patients with EGFR-mutant NSCLC treated with erlotinib. Given that PARP inhibitors may diminish or inhibit BRCA1 expression, combining olaparib with gefitinib could potentially enhance outcomes for patients with advanced EGFR-mutant NSCLC. However, the study conducted by Garcia-Campelo et al. didn’t show a significant advantage from the combination treatment of gefitinib and olaparib (19).

To the best of our knowledge for the first time, this meta-analysis aims to association between olaparib monotherapy and combination therapy with other agents like durvalumab for lung cancer.

## 2 Methods

In this systematic review and meta-analysis, we intended to specify the treatment of lung cancer by olaparib as monotherapy to combination of olaparib plus gefitinib, and combination of olaparib plus durvalumab. Our methodology cohere to the PROSPERO guidelines (International Prospective Register of Systematic Reviews). The research protocol of this review was registered to Open Science Framework (OSF) (https://api.osf.io/v2/nodes/3748c/?version=2.20).

### 2.1 Search strategy

An advanced literature search was performed up to August 13, 2023 to replevy applicable articles from following databases: Pubmed, Scopus and Google Scholar. The search strategy contained four main subgroups of keywords. The subgroups involved terms related to lung cancer, olaparib for monotherapy, gefitinib and durvalumab for combination therapy, as well. The subgroups were collaborated using the ‘AND’ operator, and no restrictions were applied concerning the date, publication type, or language. The search strategy was adjusted according to the format of query for each database. To make sure all the related articles were included, we screened the reference lists of applicable systematic reviews and included studies that were obtainable in our study. All steps were independently performed by two reviewers, and any controversy were resolved through discussion between the reviewers.

### 2.2 Inclusion and exclusion criteria

The following criteria were considered in order to select the papers for our meta-analysis study:

1. Observational methodology (in order to exclude the invalidate effect of any intervention).
2. The main goal was to compare olaparib as monotherapy to combination of olaparib plus gefitinib, and combination of olaparib plus durvalumab.
3. Study population consisted of patients suffering from lung cancer.

Studies that used other types of methodology, were executed on animal models, or were performed in cellular and molecular level, and commentary or editorial ones were excluded, and studies including interventional and observational methods plus systematic reviews were included.

### 2.3 Data extraction and quality assessment

Two independent reviewers appraised each study’s title and abstract to dispose its suitability for inclusion in this meta-analysis. Studies that didn’t fulfill our criteria were excluded. The full texts of the existing studies were screened and suitable studies entered the data extraction process. Afterwards, the following items were derived for extraction in four sets: 1. Study characteristics (i.e. authors, year,location, and type of study); 2. patient-specific factors (i.e. the eligibility criteria for patients suffering lung cancer); 3. Study Design (i.e. number of participants, method and period of drug administration, proper follow-up of the patients, technique used to evaluate the patients’ response to relative therapies); 4. Outcomes (i.e. progression free survival of the patients). Then, our reviewer used the critical appraisal checklists for Randomized Control Trial studies developed by the Joanna Briggs Institute (JBI) (https://jbi.global/critical-appraisal-tools). Another author assisted in the process in case of disparity.

### 2.5 Statistical analysis

We used STATA 13.1 software, developed by StataCorp LP in College Station, TX, USA, for our data analysis. Results were reported as pooled odds ratios (ORs) with a 95% confidence interval, visualized in a forest plot. We evaluated heterogeneity among the eligible studies using the I2 statistic (20) and used the random effects model when significant heterogeneity was detected (I2 > 50%) (21). Furthermore, we organized a sensitivity analysis and no paper was excluded. Finally, to explore the potential for publication bias, we applied visual inspection of funnel plot symmetry and Egger’s regression analysis (22).

## 3 Results

### 3.1 Study selection

**Figure 1:**
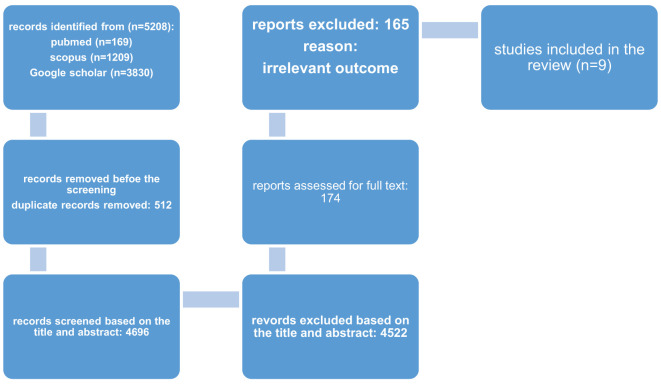
PRISMA flow diagram for current systematic.

After searching in (PubMed, Google Scholar, and Scopus) databases total of 5208 number articles were obtained, and 512 duplicates were Removed. After reviewing the title & abstract screening 174 studies remained. The final review includes 9 articles of the final full-text results, the rest of which had unrelated data were deleted. The study selection process is outlined in **Figure 1**.

### 3.2 Study characteristics

A summary of the included studies is given in **Table 1**. The trial characteristics of the eight included studies are summarized in the table below. Briefly, the trials were published between 2020 and 2023 and included 595 participants in RCTs (226 in the olaparib monotherapy group and 369 participants in the combination therapy group). The mean age of participants ranged from 36 to 89 years. The intervention duration in all RCTs was 0.3-67.1 weeks for olaparib and 4.0-63.0 weeks for durvalumab, and the dose administered ranged from 200 mg BID to 300mg BID (200mg TDS) for olaparib and 1500 mg IV for durvalumab or 250mg gefitinib. The included studies were from the UK, USA Spain, Korea, China, And France countries.

**Table 1:** Characteristics of included studies.

Author/reference	Country	Year	Study design	Participants	Sex (Fe)	Mean age	Intervention	Duration	Quality Assessment
Fennell et al. (18)	UK	2020	Randomised, double-blind, placebo-controlled, parallel arm, phase 2 trial	Patients had advanced (stage IIIB/IV) SCLC NSCLC, and had to be chemo-naive	1. Olaparib group (N=32): (Male=50%) (Female=50%) 2. Placebo group(N=38): (Male=63%) (Female=37%)	Age - median (IQR): 1. Olaparib group: 65 (61−72) 2. Placebo groupe: 63 (59−70)	The initial study dose of oral olaparib or placebo tablets was 300mg administered twice daily in 21-day cycles until disease progression, unacceptable toxicity, or patient withdrawal of consent. Dose interruption was allowed for any grade of toxicity for a maximum of 14 days until complete recovery or reversion to Common Terminology Criteria for Adverse Events(CTCAE) version 4.03 grade 1. Dose reductions were allowed to 250mg twice daily (dose level -1) or 200 mg twice daily (dose level -2). Dose escalations were not permitted	Between 27th August 2014 and 14th November 2017	12/13
Garcia-Campelo et al. (19)	Spain & Mexico	2020	Randomized phase IB/II study	Patients with treatment-naïve, pathologically confirmed stage IV NSCLC, with centrally confirmed EGFR mutations and measurable disease	Arm A: gefitinib (N=91): Female 62.64% Arm B: gefitinib plus olaparib (N=91): Female 72.53%	Arm A: gefitinib (N=91): 68 (36-85) Arm B: gefitinib plus olaparib (N=91): 65 (39-85)	182 patients underwent randomization, 91 received gefitinib and 91 received gefitinib plus olaparib. Patients were randomly allocated (1:1) to receive gefitinib 250 mg daily or gefitinib 250 mg daily plus olaparib 200 mg three times daily in 28-day cycles. The primary endpoint was PFS. Secondary endpoints included overall survival (OS), response rate, safety and tolerability	3 years (Between September 2013, and July 2016)	13/13
Desmond et al. (23)	USA	2022	An expansion cohort of a proof-of-concept phase I/II study (NCT02484404)	Fifteen individuals with advanced NSCLC who had received at least one prior platinum-based regimen.	—	Median age: 66 years	Eligible participants were enrolled in an expansion cohort of a proof-of-concept phase I/II study and received O 300 mg PO BID with D 1500 mg IV every 4 weeks until disease progression or unacceptable toxicity. The primary end-point was PFS	Between July 2016 and July 2021	9/13
Krebs et al. (24)	UK	2023	A phase 1/2, open-label, single-arm, multicenter, international basket study	Patients with histologically or cytologically confirmed relapsed limited or extensive-stage SCLC.	Patients (n = 38): Male= 55.3%	Patients (n = 38): Median (range)= 62.5 (44–76)	Patients with previously treated limited or extensive-stage SCLC received oral olaparib 300 mg twice daily, as run-in for 4 weeks, then with durvalumab (1500 mg intravenously every 4 weeks) until disease progression. As previously described for breast cancer, all patients in the SCLC cohort received olaparib monotherapy (300 mg, oral tablet, twice daily) for 4 weeks, followed by a combination of olaparib (300 mg orally twice daily) and durvalumab (1500 mg, intravenously), administered every 4 weeks (28-day cycle) until disease progression or intolerable toxicity.	Median total treatment duration (range) was 12.1 weeks (0.3–67.1 weeks) for olaparib and 8.0 weeks (4.0–63.0 weeks) for durvalumab. Median investigator-assessed duration of response was 3.6 months (IQR, 2.6–4.6 months)	12/13
Huang et al. (25)	China	2023	TRIDENT is a single arm, multicenter, phase 2 study	Patients with treatment-naïve ES-SCLC aged >18 with ECOG PS 0-2	—	—	Durvalumab (1500 mg) was concurrently administered with platinum-etoposide every 3 weeks for 4 cycles, followed by durvalumab 1500 mg every 4 weeks plus olaparib 300 mg twice daily until disease progression or unacceptable toxicity	Between August 2021 and August 2022. At the data cutoff on August 9, 2023, the median duration of follow-up was 13.0 months	11/13
Woll et al. (26)	UK	2022	Randomised, double-blind, placebo-controlled, phase II trial,	Patients with pathologically confirmed SCLC (including limited and extensive stage disease) and had achieved a complete or partial response after at least three cycles of first line chemotherapy with etoposide and either cisplatin or carboplatin	Placebo: Male=46% Olaparib BD: Male=49% Olaparib TDS: Male=42%	Placebo (N = 74): Median (Range) = 64 (43-86) Olaparib BD (N = 73): Median (Range) = 66 (43-89) Olaparib TDS (N = 73): Median (Range) = 63(42-82)	Patients received oral olaparib 300 mg BD or 200 mg TDS or matching placebo in continuous 28 day cycles for two years or until disease progression, death, unacceptable toxicity, or withdrawal of patient consent	Between 21 November 2013 and 11 December 2015	
Postel-Vinay et al. (27)	France & Spain	2023	Randomized double-blind phase II trial		Arm Placebo (N = 27): Male= 85% Arm olaparib (N = 33): Male= 82%	Arm Placebo: Median (range) = 65 (47-82) Arm olaparib: Median (range) = 62 (53-86)	Patients initially received the standard-of-care: four to six 21-day cycles of any platinum-doublet therapy, excluding taxane-based doublets. Patients with partial or complete response (based on RECIST v1.1) after 4–6 cycles of platinum-based chemotherapy were randomly assigned, in a one-to-one ratio, to receive maintenance olaparib or placebo. Olaparib or placebo was administered orally, at a dose of 300 mg twice a day in 28-day cycles, and started no later than 6 weeks after the last administration of chemotherapy. Treatment was administered until disease progression or unacceptable toxicity. Crossover to olaparib was not allowed	The median duration of follow-up was 39.3 months [CI 95%: 27.3–46.0]	13/13
Park et al. (28)	Republic of Korea	2023	This study was designed as a biomarker‐driven umbrella trial called SUKSES (Small Cell Lung Cancer Umbrella Korea Studies) as previously reported.	Patients with SCLC and SUKSES‐S experienced disease progression during or within 6 months after first‐line platinum‐based chemotherapy. Patients who relapsed >6 months after the last dose of first‐line therapy were allowed to participate in the trial after second‐line platinum chemotherapy (with participation in SUKSES as third‐line therapy). All patients had an Eastern Clinical Oncology Group Performance Score of 0 or 1, normal organ and bone marrow function, and a life expectancy of ≥16 weeks	SUKSES‐B (olaparib, n = 15): Male = 93.3% SUKSES‐N2 (olaparib and ceralasertib, n = 26): Male = 92.3%	SUKSES‐B: Median (range) = 68 (48–76) SUKSES‐N2: Median (range) = 66 (47–78)	In the olaparib monotherapy arm, olaparib 300 mg by mouth was administered every 12 hours with approximately 240 mL of water. Each cycle of treatment was 21 days in duration. A computed tomography scan was conducted every 6 weeks for response evaluation. In the olaparib and ceralasertib combination arm, the same dosing schedule of olaparib was performed but with the addition of ceralasertib at 160 mg by mouth, every day from day 1 to day 7 of each cycle. One cycle of treatment was 28 days, and a computed tomography scan was conducted every 8 weeks	From August 2016 to December 2020. The median follow‐up duration of the olaparib monotherapy arm was 8.1 months (95% CI, 5.1–16.5). The median follow‐up duration of the olaparib and ceralasertib combination arm was 7.2 months (95% CI, 6.0–10.2).	
Karachaliou et al. (29)	Spain	2020	A phase 1B and 2B study treatment	Patients with metastatic EGFR-mutant NSCLC	Gefitinib (N = 51): Male = 35% Gefitinib + Olaparib (N = 40): Male = 25%	Gefitinib: median age (range) = 70 (36-85) Gefitinib + Olaparib: median age (range) = 65 (39-85)	In the phase 1B dose escalation part of the study, tolerance in the absence of pharmacokinetic interactions and the activity of gefitinib plus olaparib were confirmed in 22 patients with EGFR-mutant NSCLC. The recommended phase 2 dose was 250 mg of gefitinib once daily plus 200 mg of olaparib three times daily	Between July 2013 and July 2016. PFS and OS were evaluated at the final data cutoff point on July 2017	12/13

### 3.3 Meta-analysis

**Figure 2:**
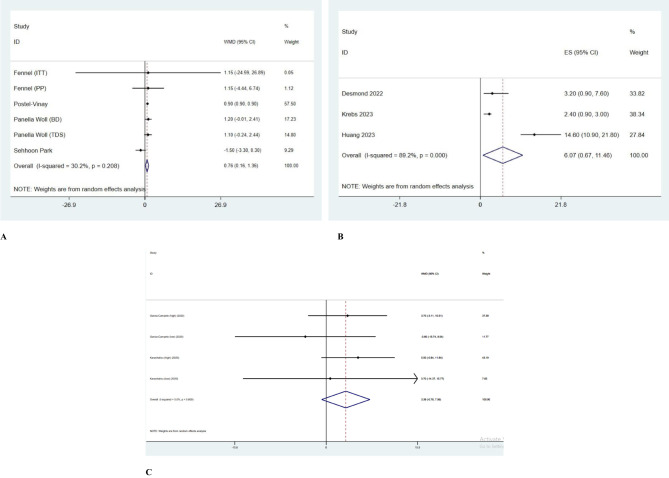
**(A)** Forrest plot of olaparib monotherapy. **(B)** Forrest plot of olaparib and durvalumab combination therapy. **(C)** Forrest plot of olaparib and gefitinib combination therapy.

WMD levels were reported in 7 included studies (**Figure 1**). Compared to baseline, olaparib monotherapy increased PFS level [ES= 7.76; 95% CI= 0.16 to 1.36; P=0.208]; however, between-study heterogeneity was reported low (I2 = 30.2%). Olaparib maintenance therapy increased PFS compared to placebo in platinum-sensitive NSCLC patients [ES= 0.9; 95% CI= 0.9 to 0.9]. Also, the obtained results indicate that olaparib as maintenance treatment, both in the form of BD [ES= 1.20; 95% CI= -0.01 to 2.41] and in the form of TDS [ES= 1.10; 95% CI= -0.24 to 2.44], in patients with chemosensitive SCLC didn’t create a statistically significant difference in improving PFS or OS, and more studies are needed in this regard (**Figure 2A**).

Using olaparib, either alone or in combination with ceralasertib, couldn’t achieve the predefined efficacy endpoint. Nevertheless, there was a noticeable increase in disease stabilization within the combination treatment group. To enhance efficacy, further exploration of olaparib in SCLC is warranted [ES= -1.50; 95% CI= -3.30 to 0.30].

Although between-study heterogeneity was high (I2 = 89.2%), combination therapy with durvalumab and olaparib decreased PFS level compared to the olaparib group according to 3 studies [ES=6.07; 95% confidence interval (95% CI) = 0.67 to 11.46; P=0.000] (**Figure 2B**).

The effect of combination therapy with gefitinib and olaparib on PFS was reported in 2 studies. Adding gefitinib to olaparib decreased PFS compared to olaparib only group, significantly (ES=3.39; 95% CI=-0.78 to 7.56; P=0.609); however, between-study heterogeneity was low (I2 = 0.0%; **Figure 2C**).

### 3.4 Risk of bias of included studies

The methodological quality of included studies was assessed using the JBI tool. All of the studies demonstrated excellent quality. Funnel plot was symmetric with pseudo 95% confidence limits and the study was not biased.

## 4 Discussion

This meta-analysis investigated the efficacy of olaparib in NSCLC. A total of 518 patients from seven studies were included. Olaparib demonstrated a significant improvement in PFS when administered as a monotherapy. However, due to limited data, the efficacy of olaparib in combination with gefitinib or durvalumab for NSCLC remains inconclusive. Further research is warranted to elucidate the potential benefits of these therapeutic regimens.

While clinically approved PARP inhibitors — including olaparib, niraparib, rucaparib, talazoparib, and veliparib — demonstrably block PARP1 and PARP2 with similar efficacy, their capacity to induce PARP trapping varies significantly. These disparities in trapping potency are considered a key factor underlying the differing dosage guidelines for these agents, as heightened PARP trapping correlates strongly with severe myelosuppressive effects (30, 31).

In 2018, olaparib gained approval for treating BRCA-mutated HER2-negative metastatic breast cancer. This progress continued with its approval for pancreatic cancer in 2019 and metastatic castrate-resistant prostate cancer in 2021 (32).

A randomized trial of 91 patients with EGFR-mutant NSCLC evaluated the impact of BRCA1 mRNA expression on PFS when treated with olaparib plus gefitinib or gefitinib alone. Patients with high BRCA1 mRNA expression exhibited significantly longer PFS in the combination group (12.9 months) compared to the gefitinib-only group (9.2 months). This effect was more pronounced in patients with high BRCA1 levels. Conversely, patients with low BRCA1 levels had longer PFS when treated with gefitinib alone. Additionally, low CTLp mRNA levels, a subtype of BRCA1 C complexes, were associated with prolonged PFS in EGFR-mutant patients receiving olaparib plus gefitinib (29).

The GOAL study, a randomized, phase IB/II trial, evaluated the efficacy of olaparib combined with gefitinib compared to gefitinib alone in 182 patients with EGFR-mutant NSCLC. While the combination group demonstrated higher response rates and longer durations of response, there was no significant difference in median PFS between the two groups (10.9 months vs. 12.8 months). Previous research has linked low BRCA1 mRNA levels to improved PFS in patients with EGFR-mutant NSCLC treated with erlotinib (19). It is hypothesized that adding olaparib to gefitinib might enhance treatment outcomes in EGFR-mutant advanced NSCLC by inhibiting BRCA1 expression (19, 29).

The PIPSeN trial, a phase 2 randomized study, investigated the use of maintenance olaparib in 60 patients with platinum-sensitive NSCLC. While patients in the olaparib group experienced a slightly longer median PFS (2.9 months) compared to the placebo group (2 months), there was no significant difference in overall survival (OS) between the two groups (9.4 months vs. 9.5 months). Due to early termination, this study was underpowered to detect significant differences. Platinum sensitivity is a biomarker associated with PARP inhibitor sensitivity. Therefore, evaluating olaparib in platinum-sensitive advanced NSCLC patients as a PARP inhibitor is warranted (18).

The MEDIOLA study, an open-label, phase 1/2 basket trial, evaluated the efficacy of olaparib plus durvalumab in relapsed SCLC. Although 40 patients were enrolled, only 38 were assessed for efficacy. The prespecified target of a 12-week disease control rate (DCR) was not met. However, in the pretreated SCLC population, median overall survival (OS) was promising. Given the potential for PARP inhibitors to enhance antitumor activity and the potential synergy with immune checkpoint inhibitors, further exploration of this combination in relapsed platinum-sensitive SCLC is warranted (24).

The TRIDENT trial, a single-arm, phase 2 study, evaluated the efficacy of olaparib plus Durvalumab as maintenance therapy in patients with extensive-stage (ES)-SCLC who had received first-line treatment with Durvalumab plus chemotherapy. The combination demonstrated promising antitumor activity without any new safety concerns. PARP inhibitors are known to modify tumor immunogenicity, exhibit antitumor activity, and can increase sensitivity to anti-PD-1/PD-L1 therapies. These characteristics suggest that PARP inhibitors may be valuable in combination with immune checkpoint inhibitors like durvalumab for ES-SCLC patients (33).

In an expansion cohort of a phase II study, the combination of olaparib plus durvalumab was evaluated in 15 patients with advanced, previously treated NSCLC. While a modest efficacy was observed overall, patients with high PD-L1 expression (>50%) and prior immune checkpoint inhibitor (ICI) therapy demonstrated a trend towards longer PFS, although this difference was not statistically significant. Preclinical research suggests that PARP inhibitors can potentially enhance the response to ICIs due to their immunostimulatory effects (23).

A study conducted by Thomas et al. found that combining PARP inhibitors with durvalumab immunotherapy could lead to a notable increase in PFS (over 5 months); however, the result wasn’t statistically significant (34). A comparable study was carried out four years later by Krebs et al., which found that combining PARP inhibitors with durvalumab immunotherapy showed no significant difference in OS and PFS, with median values of 2.4 and 7.6, respectively, when compared to other therapy combinations (24).

Unlike breast or ovarian cancer, which have a relatively high BRCA mutation rate, BRCA mutations in SCLC were found in less than 3% of the population (35). A promising method for selecting suitable SCLC patients for PARP inhibitors therapy involves the measurement of biomarkers. Among the most extensively studied is SLFN11, a biomarker whose expression levels are linked to sensitivity to DNA-damaging therapies. SLFN11 is a DNA or RNA helicase that is recruited to stalled replication forks when single-strand breaks or double-strand breaks occur during the intra-S phase checkpoint. It plays a key role by disrupting homologous recombination repair, which leads to cell cycle arrest and eventually cell death (36). Higher levels of SLFN11 have been linked to increased sensitivity to DNA-damaging chemotherapies, including PARP inhibitors, leading to improved PFS and OS in triple-negative breast cancer (37). Conversely, the absence or low expression of SLFN11 has been associated with resistance to various DNA-damaging agents, including platinum-based drugs and PARP inhibitors (38). SLFN11 was found to be significantly overexpressed, even more so than in non-small cell lung cancer, making it a potential biomarker for predicting response to PARP inhibitors in SCLC (39). Another potential marker for predicting sensitivity to PARP inhibitors is E-Cadherin. When combined with LDH measurement, it may assist in patient stratification and provide insights into the overall prognosis of SCLC patients (40, 41). However, further research is required to validate these markers and identify new ones with greater predictive value.

The reduced efficacy of combination therapy relative to monotherapy may stem from several mechanisms. First, excessive DNA damage induction that in it, combining PARP inhibitors (e.g., olaparib) with chemotherapy or radiation can exceed cellular repair capacities, causing indiscriminate cell death in both cancerous and healthy tissues. The clinical development of PARP inhibitor and platinum-based drug combinations faces complexities due to shared toxicity profiles, notably myelosuppression. A critical limitation of this approach lies in its narrow therapeutic window, stemming from the non-selective impact of both agents on healthy tissues, which exacerbates chemotherapy-induced toxicities; As happened in the BROCADE3 trial, adverse events led to study drug discontinuation (30, 42, 43). Second issue is unintended pathway activation that in it, Pairing PARP inhibitors with other DNA-damaging therapies may trigger alternative repair mechanisms (e.g., non-homologous end joining), circumventing the targeted cell death mechanism (synthetic lethality). Combining PARP inhibitors with tumor-infiltrating lymphocytes — harvested from tumors and cultured outside the body — may offer a viable therapeutic strategy for triple-negative breast cancer. Nevertheless, studies indicate that PARP inhibitors use may inadvertently increase PD-L1 levels, amplifying immunosuppressive mechanisms within the tumor microenvironment (44).

Pharmacokinetic interactions may further complicate combination approaches. For instance, olaparib is primarily processed through the CYP3A4/5 enzymatic pathway, meaning drugs that boost CYP3A activity lower plasma concentrations of olaparib, whereas CYP3A inhibitors elevate drug exposure. Additionally, overlapping toxicities may pose some challenges; PARP inhibitors (associated with blood cell deficiencies and fatigue) interact adversely with chemotherapies (e.g., drugs causing bone marrow suppression) or immunotherapies (e.g., liver toxicity from checkpoint inhibitors), often requiring dose reductions that undermine therapeutic efficacy. In patients with previously untreated metastatic nonsquamous NSCLC lacking actionable genetic mutations, the combination of pembrolizumab and maintenance olaparib showed no significant improvement in PFS or OS when compared to pembrolizumab paired with pemetrexed-based chemotherapy (45, 46).

Designing clinical trials for PARP inhibitor-chemotherapy combinations involves navigating variables such as drug characteristics (e.g., pharmacokinetics), combination partners, dosing protocols, target patient groups, and evolving regulatory standards. For instance, the FDA’s approval of cisplatin/paclitaxel/bevacizumab hindered veliparib-based regimen development. Regulatory approval requires demonstrating the combination’s superiority over individual agents, supported by robust preclinical evidence. Success demands sustained collaboration among researchers, sponsors, and patients, along with flexibility to adapt to shifting clinical environments (42).

This study presents a comprehensive analysis of three different treatment regimens for lung cancer: olaparib monotherapy, olaparib plus gefitinib, and olaparib plus durvalumab. As the first systematic review and meta-analysis on this topic, our findings provide a valuable initial assessment. Future research is warranted to further investigate the efficacy and safety of these regimens, both individually and in combination. Because of the few results have been obtained, it isn’t possible to give a definitive opinion; Therefore, it is necessary to do more studies. While our study is the first of its kind, it is limited by the relatively small number of studies available and the lack of significant improvements in PFS with the current combination therapies. Despite these limitations, the findings can be extrapolated to other populations.

## 5 Conclusion and implications

Combining PARP inhibitors with chemotherapy represents an emerging yet complex strategy aimed at amplifying the anticancer efficacy of both therapies, potentially broadening their use to more patients. Our analysis demonstrated that olaparib monotherapy can improve PFS in patients with lung cancer. However, we couldn’t find significant benefits with the combination of olaparib and gefitinib or olaparib and durvalumab. Given the heterogeneity and limited number of studies, larger and more robust trials are needed to evaluate the efficacy of these regimens in improving PFS and treating lung cancer. Despite the success of PARP inhibitors in cancers with DNA repair defects, personalized patient selection remains crucial. Advances in multi-omics and ongoing clinical trials are poised to address these challenges, enabling tailored therapies and improved resistance management in the near future.

## Data Availability

The original contributions presented in the study are included in the article/supplementary material. Further inquiries can be directed to the corresponding author.
